# Tuning of Molecular Electrostatic Potential Enables Efficient Charge Transport in Crystalline Azaacenes: A Computational Study

**DOI:** 10.3390/ijms21165654

**Published:** 2020-08-06

**Authors:** Andrey Sosorev, Dmitry Dominskiy, Ivan Chernyshov, Roman Efremov

**Affiliations:** 1 Department of Structural Biology, Shemyakin-Ovchinnikov Institute of Bioorganic Chemistry of the Russian Academy of Sciences, 117997 Moscow, Russia; r-efremov@yandex.ru; 2 Molecular Spectroscopy Department, Institute of Spectroscopy of the Russian Academy of Sciences, 108840 Moscow, Russia; 3 Faculty of Physics and International Laser Center, Lomonosov Moscow State University, 119991 Moscow, Russia; di.dominskiy@physics.msu.ru; 4 ChemBio Cluster, ITMO University, 191002 Saint Petersburg, Russia; ivan-chernyshoff@yandex.ru

**Keywords:** organic electronics, organic semiconductors, molecular design, crystal design, π-stacking, charge mobility

## Abstract

The chemical versatility of organic semiconductors provides nearly unlimited opportunities for tuning their electronic properties. However, despite decades of research, the relationship between molecular structure, molecular packing and charge mobility in these materials remains poorly understood. This reduces the search for high-mobility organic semiconductors to the inefficient trial-and-error approach. For clarifying the abovementioned relationship, investigations of the effect of small changes in the chemical structure on organic semiconductor properties are particularly important. In this study, we computationally address the impact of the substitution of C-H atom pairs by nitrogen atoms (N-substitution) on the molecular properties, molecular packing and charge mobility of crystalline oligoacenes. We observe that besides decreasing frontier molecular orbital levels, N-substitution dramatically alters molecular electrostatic potential, yielding pronounced electron-rich and electron-deficient areas. These changes in the molecular electrostatic potential strengthen face-to-face and edge-to-edge interactions in the corresponding crystals and result in the crossover from the herringbone packing motif to π-stacking. When the electron-rich and electron-deficient areas are large, sharply defined and, probably, have a certain symmetry, calculated charge mobility increases up to 3–4 cm^2^V^−1^s^−1^. The results obtained highlight the potential of azaacenes for application in organic electronic devices and are expected to facilitate the rational design of organic semiconductors for the steady improvement of organic electronics.

## 1. Introduction

Organic semiconductors with efficient charge transport, i.e., high charge mobility *μ*, are in great demand for organic electronics. However, only several organic semiconductors (OSs) with *μ* > 1 cm^2^ V^−1^ s^−1^ have been discovered [1,2]. Nevertheless, the tunability of the molecular structure of OSs [1] gives hope for finding novel high-mobility materials among them. Unfortunately, the search for high-mobility OSs still relies mainly on the trial-and-error approach, and design rules that could direct and rationalize this search are highly desirable.

OSs consist of π-conjugated molecules held together by weak intermolecular forces [3,4]. The molecular nature of the OSs limits the charge transport in these materials because although charge carriers (electrons or holes) are efficiently delocalized within the molecules, their intermolecular delocalization or transfer from one molecule to another is generally hindered by the weakness of the electronic interaction between the molecules [3,4]. Specifically, local and non-local electron–vibrational (electron–phonon) interaction, quantified by reorganization energy *λ* and lattice distortion energy, respectively, overwhelm the intermolecular electronic interaction quantified by the charge transfer integrals *J*, and induce charge localization. As a result, charge transport proceeds via an inefficient hopping mechanism; the hopping rate increases with *J* and decreases with *λ* [5,6,7]. Nevertheless, if *J* is sufficiently large, significant intermolecular charge delocalization, resulting in efficient bandlike charge transport, can take place [5,8,9]. Thus, increasing *J* and decreasing *λ* is important for the improvement of charge mobility in OSs.

The *λ* values can be readily predicted from just the OS molecular structure [9]. On the contrary, the prediction of *J* values from just the molecular structure seems to be not feasible yet. Indeed, the *J* values are determined by the relative positions of the molecules and are very sensitive to the latter [10]. Thus, the prediction of *J* requires reliable modeling of molecular arrangement; for crystalline OSs, to which record-*μ* OSs belong and which we will focus on, this means the prediction of the crystal structure. Unfortunately, reliable crystal structure prediction for OSs is not straightforward and remains hardly feasible [11,12,13], precluding the desired assessment of *J* and hence *μ* just from the molecular structure. In this context, semiempirical “rules of thumb” linking the molecular structure, crystal structure and charge transport properties are of significant importance.

The formation of molecular crystals is guided by the avoidance of a vacuum and the lessening of repulsive intermolecular interactions, as well as the boosting of attractive molecular forces [11]. There are two major types of molecular packing motifs in crystalline OSs: herringbone and π-stacking (the latter is sometimes divided into slipped stack and brick wall arrangements [1]). In herringbone-type crystals, the molecular planes of the adjacent molecules are tilted with respect to each other due to the preference for edge-to-face CH⋯π interactions over face-to-face π⋯π and edge-to-edge H⋯H ones. This is maintained by the electrostatic interaction between slightly electron-deficient (bearing partial positive charge) hydrogen atoms and an electron-rich (bearing partial negative charge) π-conjugated core [6,14]. In the π-stacking packing motif, the molecules are arranged in parallel—in the face-to-face manner—and π-conjugated systems of the molecules can significantly overlap. This packing is realized if face-to-face and edge-to-edge interactions overwhelm edge-to-face interactions [15] due to either altered electrostatics or steric hindrance induced by bulky side groups [16,17,18]. It was noticed that the largest *J* is generally observed for face-to-face arrangements typical of the π-stacking packing motif [13,19]. For instance, in the OSs with record *μ*, e.g., crystalline rubrene (hole transport) [20] and F_2_-TCNQ (electron transport) [21], the largest *J* is observed for the face-to-face orientation of adjacent molecules and amounts to ~70–110 meV, which is significantly larger than the energy of thermal fluctuations (25 meV at room temperature) [8]. For this reason, crystalline OSs with π-stacking have attracted particular attention in recent decades.

One of the strategies for designing crystals with π-stacking is the introduction of electron-withdrawing groups (EWGs), especially cyano-groups and halogen (e.g., fluorine) atoms. There are two reasons for the structural changes induced by these groups. The first reason is the strengthening of edge-to-edge interactions due to CH⋯EWG contacts, e.g., CH⋯F and CH⋯NC [15]. The second reason is the enhancement of face-to-face π⋯π interactions due to the formation of electron-deficient and electron-rich parts in a π-conjugated system as a result of electron density redistribution [17,19]. Note that the introduction of EWGs can sometimes result in the opposite structural changes, as extensive fluorination can induce the crossover to a herringbone structure from a π-stacking one [19,21,22]. The insertion of a heteroatom directly in the conjugated core, e.g., the substitution of CH with N (N-substitution), can be another way of tuning the crystal structure, as this substitution also weakens edge-to-face interactions and facilitates edge-to-edge and face-to-face interactions [13,23,24,25]. However, this molecular structure modification is much less common in the design of OSs as compared to fluorination and cyano-substitution, and its effect is worth a detailed investigation. Importantly, N-substitution could be more beneficial for charge transport than halogenation since, in the former case, “electronically insulating” hydrogen and halogen atoms are removed from the molecular structure, and N atoms bearing the π-electron density come in “conductive” contact with the π-conjugated systems of the nearby molecules [15].

A convenient playground for studying the impact of N-substitution on the crystal structure and properties is the series of oligoacenes—one of the most studied classes of OSs. Accordingly, a comparison of the oligoacenes and their N-substituted counterparts—azaacenes—was performed in several studies [13,23,24,25]. It was observed that, while oligoacenes favor herringbone crystal packing [26], their N-substituted counterparts can show a π-stacking packing motif [23,24,25,27]. In line with the reasoning provided above, changes in the crystal structure were attributed to the changes in the electrostatic potentials of the oligoacene molecules with N-substitution and the emergence of CH⋯N weak hydrogen bonds [23,24]. Nevertheless, a systematic study of the effect of N-substitution on the crystal packing of various oligoacenes is lacking. For instance, molecular packing of crystalline azaacenes with more than two nitrogen atoms have not been addressed theoretically before.

Similarly, the effect of N-substitution on the charge transport properties is also poorly studied. Oligoacenes show considerable hole mobilities [2,28]; however, electron transport in them is not observed in organic field-effect transistors (OFETs) because of the high lowest unoccupied molecular orbital (LUMO) levels preventing electron injection [2]. N-substitution decreases LUMO levels and can result in efficient electron transport [27,29]. However, according to the findings of Winkler and Houk [30], as many as seven nitrogen atoms should be introduced in the pentacene core to enable electron conductivity with commonly used electrodes. To the best of our knowledge, pentacene derivatives with a maximum of four nitrogens have been synthetized to date; luckily, they already show electron conduction [27]. We have no data concerning the tests of the lower azaacenes, e.g., azaanthracenes or azatetracenes, in field-effect devices. Thus, the theoretical assessment of the highest occupied molecular orbital (HOMO) and LUMO levels, as well as electron and hole mobility in various azaacenes, is of significant interest.

In this study, we address computationally the impact of N-substitution on the molecular properties, crystal structure and charge mobility of the most popular oligoacenes—anthracene, tetracene and pentacene. The role of the number and position of the nitrogen heteroatoms in the mentioned π-conjugated cores is systematically analyzed. Only azaacenes (without side groups) that were successfully synthesized, and for which the crystal structure is known, are considered; this distinguishes our study from recent reports where predicted crystal structures were used [23,24] or substituted azaacenes were addressed [31]. Furthermore, the molecular packing and charge transport in the crystals of azaacenes with four nitrogen atoms, which have not been addressed theoretically before, are considered. We show that N-substitution facilitates π-stacking; in several cases (but not all), these changes in the molecular packing significantly improve charge transport. The positive effect of N-substitution on the charge mobility is most pronounced when the nitrogen atoms are arranged in a centrally symmetric pattern without a plane of symmetry and form large electronegative areas. This results in large hole transfer integrals (up to 85 meV) and very large electron transfer integrals (up to 175 meV), providing theoretical hole and/or electron mobilities exceeding 1 cm^2^ V^−1^ s^−1^ for three OSs: 1,5,9,10-tetraazaanthracene (**4NA**), 6,13-diazapentacene (**2NP**) and 1,7,8,14-tetraazapentacene (**4NP**). Finally, we extend the obtained results on the nucleobases—natural π-conjugated compounds bearing multiple nitrogen atoms in the conjugated core. Our findings show that tailoring the pattern of N-substitution within the conjugated core can be used as a promising strategy for the design of efficient organic semiconductors.

## 2. Results

### 2.1. Molecules

**Figure 1:**
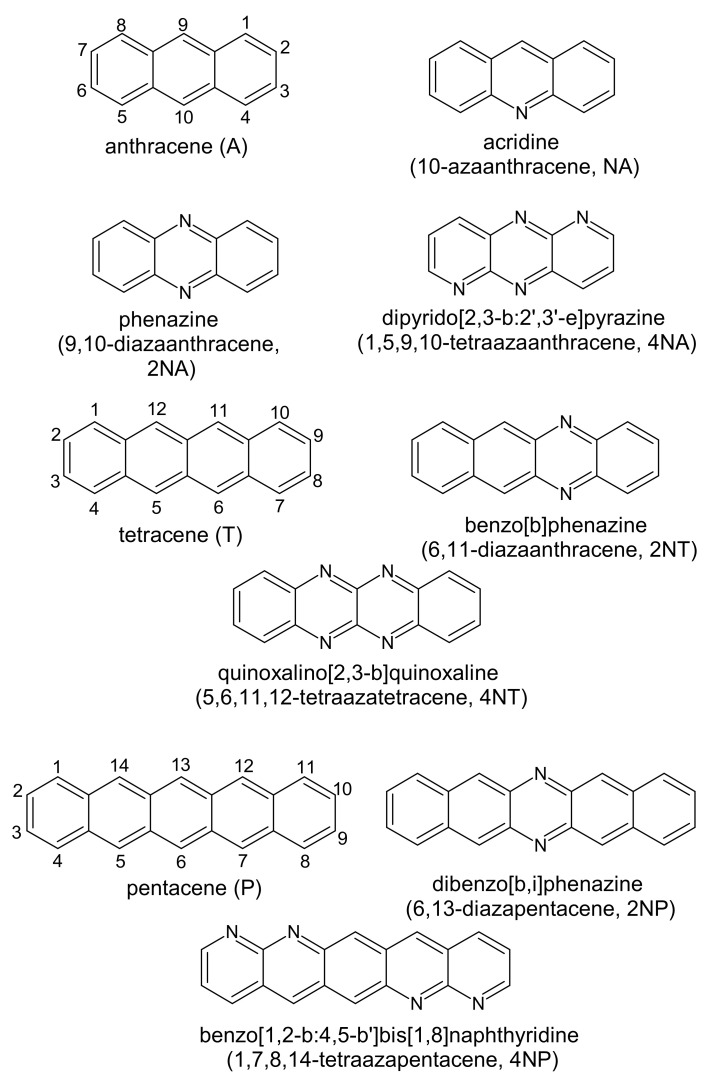
Chemical structure of the compounds studied.

**Figure 2:**
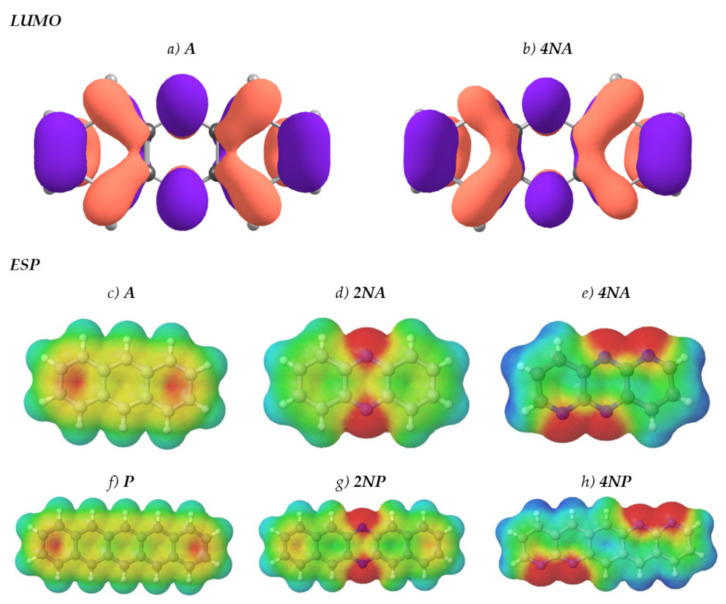
LUMO patterns (**a**,**b**) and electrostatic potential (ESP) maps (**c**–**g**) for **A** (**a**,**c**), **2NA** (**d**), **4NA** (**b**,**e**), **P** (**f**), **2NP** (**g**) and **4NP** (**h**). The purple and orange colors in panels (**a**,**b**) designate the positive and negative signs of the LUMO wavefunction. Blue color in panels (**c**-**h**) designates strongly positive ESP, and red color designates strongly negative ESP. Green and yellow colors correspond to slightly positive and slightly negative ESP, respectively.

Figure 1 presents the chemical structures of the compounds studied. These molecules fall into three series: derivatives of anthracene (**A**), tetracene (**T**) and pentacene (**P**). In each of the series, the number of nitrogen atoms varies from zero to four. Figure 2 shows LUMO patterns and electrostatic potential (ESP) maps for **A** and its nitrogen-substituted derivatives–phenazine (9,10-diazaanthracene, or **2NA**) and dipyrido[2,3-b:2′,3′-e]pyrazine (1,5,9,10-tetraazaanthracene, or **4NA**)–obtained using density functional theory (DFT). LUMO patterns and ESP maps for the other compounds studied, as well as HOMO patterns for all the compounds studied, are shown in Supplementary Information (SI), Figures S1 and S2. From Figure 2, Figures S1 and S2, it follows that N-substitution has a negligible impact on the LUMO pattern and weakly affects the HOMO pattern for all the compounds studied except for **4NA**. In the latter, the HOMO pattern is qualitatively different from all the other compounds studied. In contrast to the HOMO/LUMO patterns, the ESP pattern is dramatically affected by N-substitution, in line with previous results [12,24]. Specifically, while **A**, **T** and **P** have the electronegative center (carbon atoms) and the electropositive periphery (hydrogen atoms), the nitrogen atoms pull out the electron density, resulting in the emergence of strongly electronegative areas within the conjugated core and making the remaining part of the molecule (both carbon and hydrogen atoms) electropositive. Noteworthily, if nitrogen atoms are nearby (Figure 2e,h), the electronegative areas are large, and the ESP pattern can consist of the “stripes” of alternating positive and negative ESP. One can expect that in crystal, the molecules will pack so that areas of positive ESP for one molecule will contact the areas of negative ESP for the other molecule according to the principle of ESP complementarity [14,19]. Thus, the formed electron-rich and electron-deficient areas can direct the molecular arrangement in the crystals, as will be described below.

**Figure 3:**
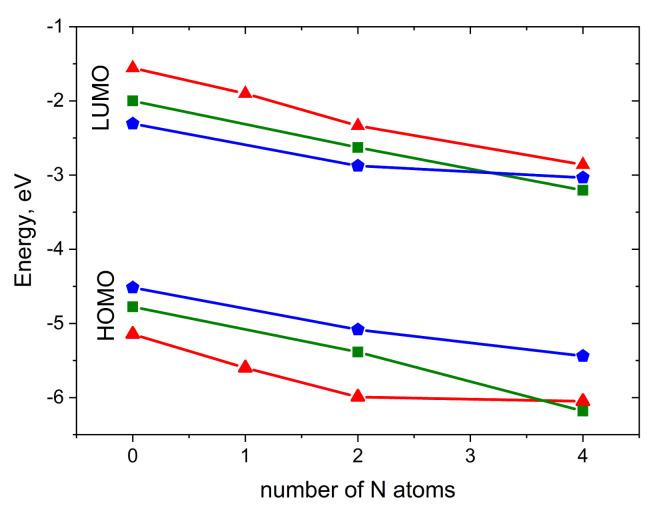
HOMO and LUMO energies for the compounds studied. Red triangles: A derivatives, green squares: T derivatives, blue pentagons: P derivatives.

Figure 3 illustrates the impact of N-substitution on the HOMO and LUMO energies of the compounds studied. As expected, N-substitution lowers both the HOMO and LUMO energies. The HOMO–LUMO gap is weakly affected by substitution; for pentacene derivatives, it is the narrowest, while for anthracene derivatives, it is the widest. For **2NP**, **4NP**, **4NT** and **4NA**, LUMO energies are low enough to enable electron injection [27,32], in line with the observation of electron conductivity in diazapentacene in [27]. In contrast, **A**, **T**, **P**, **2NT** and **2NP** are expected to maintain efficient hole injection, in line with the results of [23]. Noteworthily, **2NP** can probably show ambipolar charge transport.

### 2.2. Crystals

**Figure 4:**
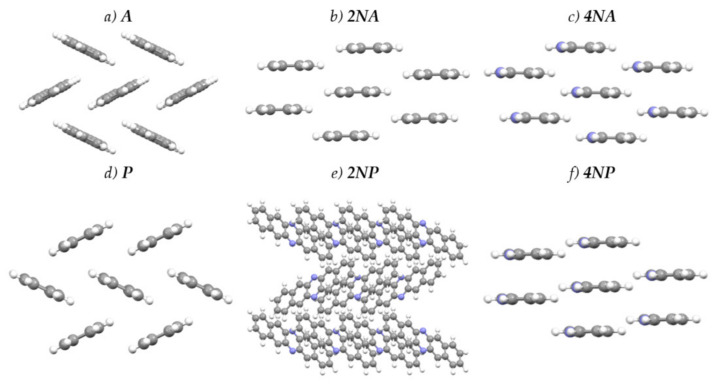
Crystal structures of the selected compounds.

**Figure 5:**
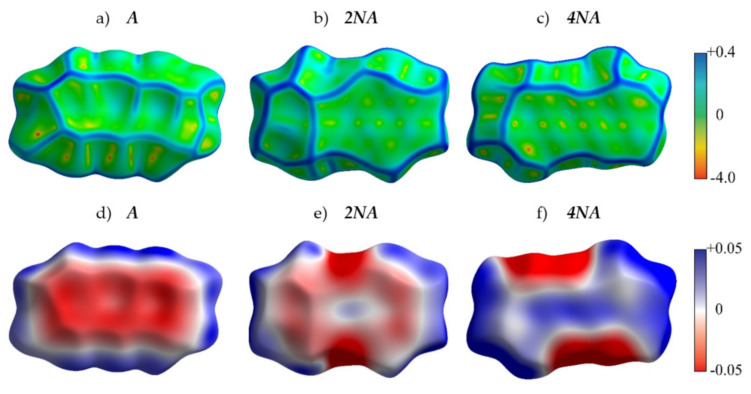
Hirshfeld surfaces of **A**, **2NA** and **4NA** mapped with curvedness (**a**–**c**) and ESP (± 65.6 kJ mol^−1^ per unit charge) (**d**–**f**). Color scale for curvedness and ESP maps is located on the right.

**Figure 6:**
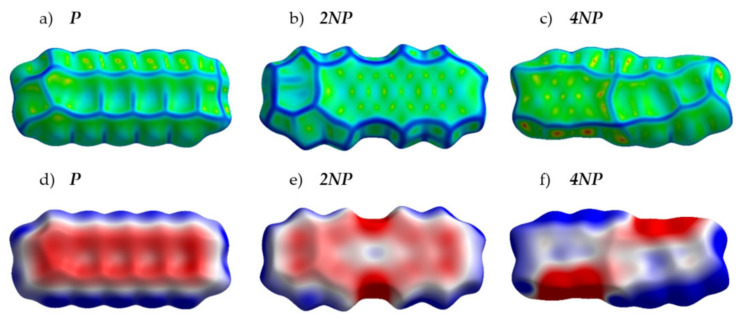
Hirshfeld surfaces of **P**, **2NP** and **4NP** mapped with curvedness (**a**–**c**) and ESP (± 65.6 kJ mol^−1^ per unit charge) (**d**–**f**). Color scheme corresponds to Figure 5.

Figure 4 presents the crystal structures of **A**, **P** and their selected N-substituted derivatives. From this figure, it follows that, while **A** and **P** show a herringbone packing motif, **2NA, 2NP, 4NA** and **4NP** exhibit π-stacking. To illustrate and analyze the impact of nitrogen atoms on the molecular arrangement, we utilized Hirshfeld surface analysis of crystal packing [33]. The Hirshfeld surface defines the space occupied by a molecule: inside this surface, the electron density from the given molecule is larger than that from the others [34]. Figure 5 and Figure 6 show the Hirshfeld surfaces for the selected crystals from **A** and **P** series, respectively (for phenazine, only one polymorph was chosen), mapped with curvedness and ESP. The corresponding Hirshfeld surfaces for **T** series and Hirshfeld surfaces mapped with other properties are shown in SI, Figures S3–S5. Curvedness maps can be used to identify planar π-stacking arrangements [33]: the latter reveal themselves by relatively large green flat regions. The map for **A** (Figure 5a) has no such regions, which is typical for herringbone packing [33]. On the contrary, the maps for **2NA** (Figure 5b) and **4NA** (Figure 5c) show one extended green region per face. This indicates π-stacking of the molecule with one adjacent molecule per face (i.e., one-dimensional π-stacking) in these crystals. A similar situation is observed for pentacene and its N-substituted derivatives: while the curvedness map for **P** (Figure 6a) has no extended flat regions, such regions are observed for **2NP** and **4NP** (Figure 6b,c), highlighting the crossover from the herringbone packing in the former crystal to the one-dimensional π-stacking in the two latter crystals. These changes are also revealed in the energy frameworks (Figures S6–S8): with N-substitution, certain (π-stacking) directions with large energy of intermolecular interaction emerge. Molecular ESPs in the crystals (Figure 5d–f; Figure 6d–f) directly inherit those of the single molecules (Figure 1c–h): introducing nitrogen atoms into the conjugated core results in the formation of extended electropositive and electronegative areas at the Hirshfeld surfaces. The latter areas facilitate electrostatic interactions between the molecules, which is also revealed in the energy framework diagrams (Figures S6–S8).

**Figure 7:**
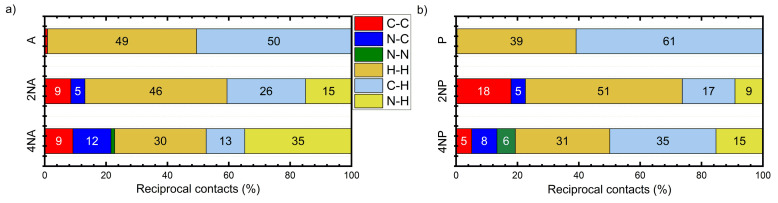
Distribution of reciprocal intermolecular contacts for selected compounds from **A** (**a**) and **P** (**b**) series arranged by molecules. “Conductive” contacts are shown in red, blue and green.

Figure 7 presents the distribution of the intermolecular contacts by the atom type for the **A** and **P** series. From Figure 7a, it follows that, in **A**, most contacts are either C⋯H or H⋯H, which is a common feature of herringbone packing. These contacts are not favorable for charge transport (“insulating”) since hydrogen atoms bear negligible electron density on the frontier orbitals (HOMO and LUMO). On the contrary, in **2NA** and **4NA**, the contacts between non-hydrogen atoms, namely C⋯C and C⋯N, emerge and their contribution increases with the number of nitrogen atoms in the molecule (Figure S9). These findings are in line with the results of previous studies for slightly N-substituted azaacenes [23,24]. C⋯C and C⋯N contacts are favorable for charge transport (“conductive”) since they enable larger *J* values due to the efficient overlap between the frontier orbitals of the adjacent molecules: both the C and N atoms can bear considerable π-electron (HOMO and/or LUMO) density. The increase in the number of “conductive” contacts with N-substitution is also observed for pentacene (Figure 7b and Figure S10) and tetracene (Figure S11) series. Interestingly, in the azaacenes with four nitrogen atoms, N⋯N contacts (which should be electrostatically unfavorable (Figures S10 and S11)) are observed. We assume that these contacts are an unintentional byproduct of the stronger attractive CH⋯N interactions: an energy decrease due to the latter contacts probably overwhelms the energy increase caused by N⋯N electrostatic repulsion. Note that azaacenes with more than two nitrogen atoms and hence extended electron-deficient and -rich areas have not addressed been theoretically before, so the effect of such heavy N-substitution on crystal packing and charge transport observed herein is more pronounced than that previously reported in [23,24,25].

### 2.3. Charge Transport

To monitor the changes in the electron and hole mobilities with N-substitution, we applied a hopping model of charge transport based on the Marcus formula for charge transfer (“hop”) rate [35]:

(1) $$k_{}=\frac{2\pi }{\hbar }J^{2}{\left(\frac{1}{4\pi \lambda k_{B}T}\right)}^{1/2}\exp\left(-\frac{{\left(\Delta E-\lambda \right)}^{2}}{4\lambda k_{B}T}\right),$$

where *ħ* is the reduced Planck constant, *k_B_* is the Boltzmann constant, *T* is the absolute temperature and Δ*E* is the electron energy difference between the initial and final sites (Δ*E* = 0 for identical molecules). Then, the charge carrier (hole or electron) diffusion coefficient was calculated by summation over all the transport directions, i.e., all the pairs of the given molecule with its neighbors and, finally, isotropic hole and electron mobilities, *μ_h_* and *μ_e_*, were calculated using the Einstein–Smoluchowski relation (see details in SI, Section S4). As follows from Equation (1), *J* and *λ* are the main parameters determining the charge transport within the hopping model. As mentioned above, the former is associated with the electronic interaction between the molecules, while the latter is determined by the electron–vibrational interaction. We will first analyze the impact of N-substitution on these parameters and then turn to its impact on *μ*.

#### 2.3.1. Reorganization Energies

**Figure 8:**
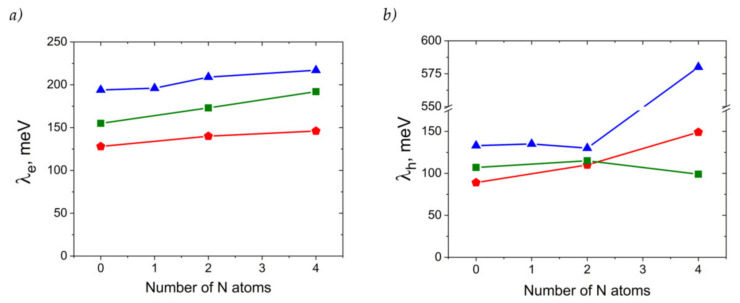
Electron (**a**) and hole (**b**) transfer reorganization energies for the compounds studied. Red triangles: **A** derivatives, green squares: **T** derivatives, blue pentagons: **P** derivatives.

Figure 8 presents the calculated reorganization energies for oligoacenes and their N-substituted counterparts. As follows from Figure 8a, reorganization energy for electron transport, *λ_e_*, increases with N-substitution. This is in line with previous results for azapentacenes [12] and could be attributed to the decrease in the non-bonding character of LUMO in N-substituted oligoacenes [36] and decreased aromaticity [37]. However, the increase is rather small (less than 25%), and is not expected to reduce the charge mobility dramatically. The small changes in *λ_e_* with N-substitution can be attributed to the small impact of the latter on the LUMO pattern. Indeed, the similar LUMO pattern of oligoacenes and azaacenes should result in a similar electron–vibrational interaction, i.e., similar *λ_e_*, since molecular vibrations are not expected to be strongly altered with N-substitution due to the comparable atomic masses of the CH group and N atom and comparable energies of the C=C and C=N bonds. Noteworthily, the small increase is probably related to the symmetric character of N-substitution, as asymmetric N-pentacenes show much larger *λ_e_* [12], probably due to the significant changes in the LUMO pattern as a result of the asymmetric insertion of heteroatoms.

Reorganization energies for hole transfer, *λ_h_*, show a more complicated behavior with N-substitution, as shown in Figure 8b. For **4NA**, *λ_h_* is more than twice as large as that for the other anthracene derivatives studied and amounts to ~580 meV (note that change in the DFT functional from B3LYP to PBE resulted in a similar *λ_h_* of ~520 meV). Such a high *λ_h_* can be attributed to the qualitatively different HOMO pattern in the **4NA** molecule than in the other OSs studied (Figure S1): this pattern nearly lacks any nodes on the bonds. On the contrary, *λ_h_* for **2NA** is lower than that for the other members of the **A** series, which can be attributed to the stronger non-bonding character of the HOMO (more pronounced nodes on the bonds) in this compound. Indeed, the larger the number of HOMO nodes on the bonds, the weaker the hole–vibrational interaction, i.e., the lower the *λ_h_* [38].

**Figure 9:**
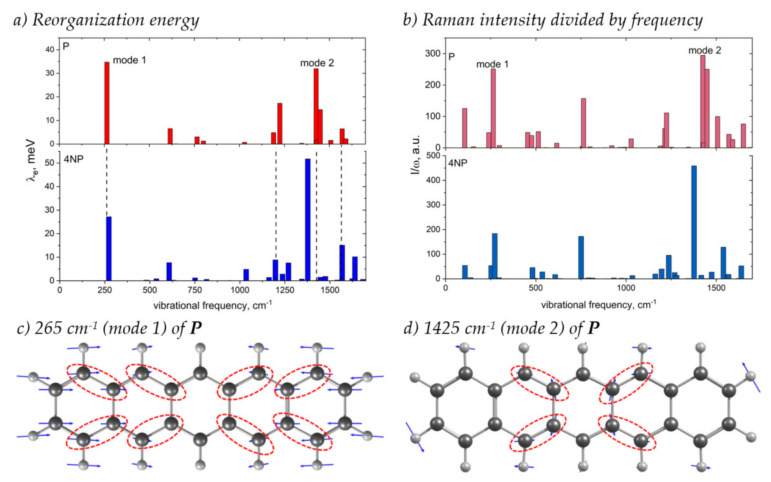
(**a**,**b**) Contribution of various vibrational modes to the electron transfer reorganization energy, *λ_i_* (**a**), and Raman intensities of these modes divided by their frequency, *I_i_*/*ω_i_* (**b**), for **P** (top) and **4NP** (bottom). (**c**,**d**) Vibrational displacements for the **P** modes showing the largest contribution to *λ* and *I*/*ω* (labeled “mode 1” and “mode 2” in panels (**a**,**b**)). Dashed ellipses highlight bonds with considerable LUMO density (Figure 2) that are significantly modulated by the corresponding vibrations. For vibrational displacements of **4NP** see Figure S12.

Figure 9 shows how the contributions of various vibrational modes to *λ_e_* change with N-substitution and compares these changes with the changes in the Raman spectrum, which was suggested to monitor the electron–vibrational interaction [39,40,41]. From this figure, it follows that the main contribution to *λ_e_* stems from two collective modes shown in Figure 9c,d: the conjugated skeleton stretching along the molecular long-axis (mode 1) and the collective C=C stretching vibrations and C–H wagging vibrations (mode 2), in line with [25]. These vibrations distort the bonds where the LUMO is localized, resulting in strong electron–vibrational coupling (Figure 2 and Figure 9c,d). With N-substitution, the frequency of mode 1 slightly increases, and that of mode 2 decreases, in line with [25]. The contribution of the latter mode to *λ* increases significantly, while that of the former decreases.

It was shown in [39] that, for a particular class of organic semiconductors—charge transfer complexes—the contribution of the vibrational mode to the reorganization energy is related to its Raman intensity: $\lambda_{i}\propto I_{i}/\omega_{i}$, where *ω_i_* is the frequency of the mode. Although the correlation between $I_{i}/\omega_{i}$ and *λ_i_* was observed for few single-component OSs as well [40,41], the generalization of this relation to the other OSs is lacking. From Figure 9b, it follows that, for both **P** and **4NP**, modes 1 and 2, which show the largest *λ_i_*, also have the largest $I_{i}/\omega_{i}$ ratio, and changes in this ratio with N-substitution (a significant increase for mode 2) resemble the changes in *λ_i_*. Moreover, from Figure 9, it follows that changes in the total *λ_e_* between **P** and **4NP** correlate with the changes in spectrally integrated $I_{i}/\omega_{i}$; both quantities increase with N-substitution. Thus, our findings highlight the potential of Raman spectroscopy for monitoring the changes in the electron–vibrational interaction with subtle changes in the chemical structure, e.g., N-substitution; however, this is the subject of a separate study.

#### 2.3.2. Charge Transfer Integrals

**Figure 10:**
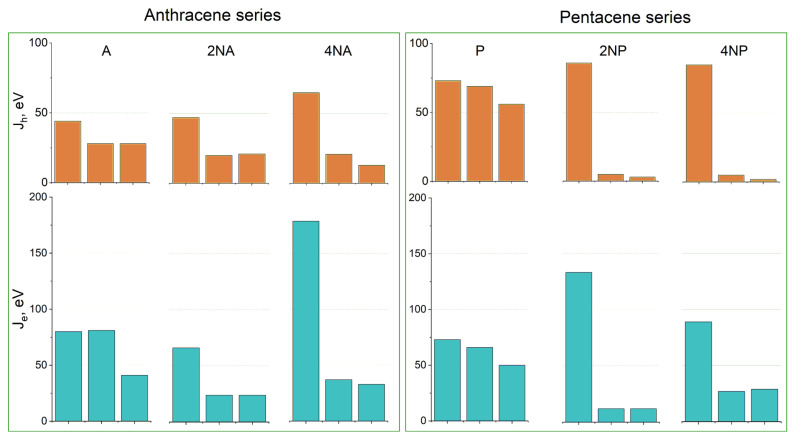
Hole (**top**, *J_h_*) and electron (**bottom**, *J_e_*) transfer integrals for the selected crystals from the (aza)anthracene and (aza)pentacene series. For each crystal, the three largest *J_h_*_,e_ values for different directions are shown.

Figure 10 shows the hole and electron transfer integrals for **A** and **P** and their selected N-substituted derivatives. From this figure, it follows that, for the **A** and **P** series, N-substitution generally results in the crossover from three directions of moderate charge transport to a single direction with efficient charge transport—the direction of face-to-face molecular arrangement (π-stacking, Figure S13)—for both holes and electrons. This is natural since face-to-face interactions are stabilized by N-substitution in the crystals studied, and π-stacking is facilitated. Moreover, similar changes in the charge transport dimensionality were observed in [19], where variations in the molecular electrostatic potential with partial fluorination induced a crossover to the π-stacking packing motif and (quasi) one-dimensional charge transport. Noteworthily, completely one-dimensional charge transport is generally detrimental for *μ* [1,2,9,42]; however, in **4NA** and **4NP**, *J_e_* (and also *J_h_* for the former compound) in the other directions amount to ~20 meV, making the charge transport not completely one-dimensional but quasi-one-dimensional. The latter regime seems to be compatible with high *μ*: for instance, in one of the most efficient p-type OSs, rubrene [2], and the decent n-type OS TCNQ [9], large *J* is also observed in one direction, and *J* values for the other directions amount to ~20 meV [8]. Noteworthily, as follows from Figure 10, the largest *J* values for the studied N-substituted anthracenes and pentacenes (except for **2NA**) are larger than those for the non-substituted counterparts. For **4NT**, all *J* values are small—there is only one direction with *J_h_* = 45 meV and *J_e_* = 13 meV—which indicates poor matching of the frontier orbitals’ phases for adjacent molecules despite their considerable spatial overlap, presumably because of poor ESP complementarity.

#### 2.3.3. Charge Mobilities

**Figure 11:**
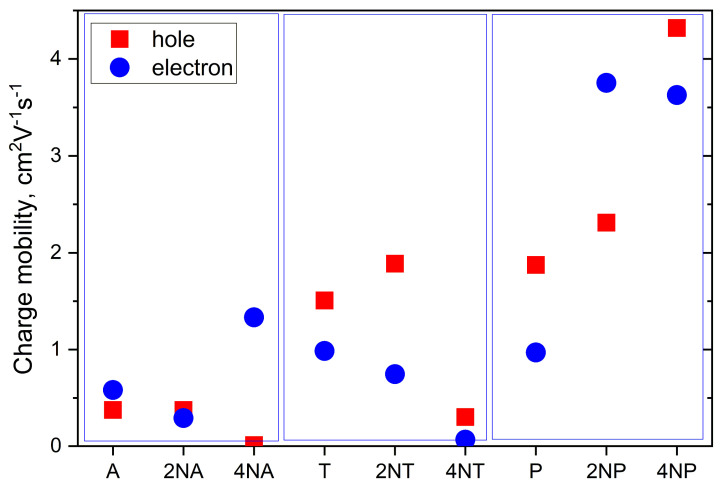
Calculated charge mobilities for the compounds studied.

Figure 11 collates the calculated hole and electron mobilities for the compounds studied. The hole mobility slightly increases as a result of the insertion of two nitrogen atoms for the **T** and **P** series, yielding *μ_h_*~1.8 and 2.3 cm^2^ V^−1^ s^−1^ for **2NT** and **2NP**, respectively, but remains nearly unaffected for **2NA** (*μ_h_*~0.4 cm^2^V^−1^s^−1^). Further N-substitution has an even more discrepant effect on *μ_h_*. Specifically, for **4NP**, *μ_h_* significantly exceeds that for **P**, amounting to a remarkably high ~4.3 cm^2^ V^−1^ s^−1^. So high *μ_h_* can be attributed to large *J_h_* (Figure 10), decent *λ_h_* (Figure 8) and significant slip of the molecules along the long molecular axis, resulting in a large distance between the molecular centers, *r*, in the direction of the most efficient transport (*r*~9 Å for **4NP** vs. *r*~4 Å for **4NP**). The latter is beneficial for the charge transport since the larger the hopping distance, the larger the *μ* (Equations ((S1)–(S2)). For **4NA** and **4NT**, *μ_h_* is lower than for **A** and **T**, respectively. Low *μ_h_* for **4NA** can be attributed to the extremely high *λ_h_* for this compound (see Figure 8), while low *μ_h_* for **4NT** can be explained by small *J_h_*.

Electron mobility decreases as a result of the insertion of two nitrogen atoms in the central ring for both **2NA** and **2NT**, but increases for **2NP**, as compared to the non-substituted counterparts. As a result, in **2NP**, *μ_e_* exceeds 3 cm^2^V^−1^s^−1^. Four nitrogen atoms result in the increase in the electron mobility for **4NA** and **4NP**, yielding *μ_e_*~1.3 and ~3.6 cm^2^V^−1^s^−1^, respectively. High *μ_e_* in the former OS can be attributed to a very large *J_e_* of ~175 meV (see Figure 10), which significantly exceeds the typical values for high-mobility OSs [2]. For **4NP**, high *μ_e_* can be explained by a decent *J_e_* of ~85 meV, the abovementioned slip of the molecules along the long molecular axis and moderate *λ_e_* (Figure 8). Noteworthily, our results were obtained within a Marcus model, which typically underestimates *μ*. Furthermore, our *μ_e_* estimate for **4NP** is larger than that we obtained in similar conditions for 6,13-bis(triisopropylsilylethynyl)tetraazapentacene (TIPS-TAP, calculated *μ_e_* = 2.7 cm^2^V^−1^s^−1^)—the TIPS-substituted tetraazapentacene with a high experimental *μ_e_* of 3.3 cm^2^V^−1^s^−1^ [27].

For the operation of OFETs and other electronic devices, not just high *μ*, but also efficient charge injection is required [43]. As follows from Figure 3, HOMO and LUMO levels are lowered with N-substitution and, hence, electron injection becomes more favorable, while hole injection becomes less favorable. This is in line with experimental data [27]; while pentacene and **2NP** showed hole (*p*-type) conductivity in OFETs, **4NP** (isomer of the compound studied herein) exhibited electron (*n*-type) conductivity. Since the LUMO levels of **4NA** and **4NT** are close to that of **4NP**, we expect that these compounds can also show electron conductivity. In conjunction with high predicted electron mobility (Figure 9), they are promising for application in n-type OFETs. In addition, **2NT** and **2NP** could show efficient hole injection and transport and outperform **T** and **P** in p-type OFETs, respectively. Finally, **2NP** and **4NP** could probably yield ambipolar mobility.

Thus, azaacenes without side groups can show very high *μ_h_* and *μ_e_*. A lack of high experimental *μ* data for these materials can be caused by the polycrystalline nature of their thin films studied in OFETs or their low HOMO/high LUMO levels (as compared, e.g., with non-substituted acenes [2] and TIPS-substituted counterparts [27]). These factors could result in an increased number of defects due to lower oxidation stability [44]. Moreover, the synthesis of **4NA** was reported very recently [45], and this compound has probably not been tested in organic electronic devices.

## 3. Discussion

Our results highlight the dramatic impact of N-substitution on the crystal structure of linear oligoacenes. This is in line with previous observations [13,14,23,25] and extends the results of the mentioned studies towards heavily N-substituted (bearing four nitrogen atoms) oligoacenes. Specifically, N-substitution facilitates π-stacking and increases the number of “conductive” C⋯C and C⋯N (and sometimes N⋯N) intermolecular contacts, instead of “insulating” H⋯H and C⋯H contacts, which dominate in oligoacenes. These structural changes can result in the increase in *J*, in line with previous observations for N-substituted oligoarenes [46]. Within the studied series of anthracene, tetracene and pentacene derivatives, *J* and *r_i_* values determine the charge mobilities, while *λ* remains comparable (except for the case of hole transport in **4NA**, see Figure 8).

Surprisingly, our results show that facilitated π-stacking does not necessarily increase charge mobility (Figure 5, Figure 6, Figure 7 and Figure 11). Nevertheless, **4NA** and **4NP**—compounds where N-substitution is heavy and its pattern has a center of symmetry but lacks a plane of symmetry—show either the largest electron or the largest hole mobilities in the **A** and **P** series, respectively. We suggest that this is because such symmetry of N-substitution results in pronounced and large electron-rich and electron-deficient “stripes”, which facilitate efficient π-stacking and favorable HOMO and/or LUMO overlap. Additionally, **4NT**, where the pattern of N-substitution has both a center and a plane of symmetry, shows low *μ_e_* and *μ_h_* due to small *J*, probably dictated by the mismatch of the HOMO and LUMO phases. Thus, we suggest that molecules with a considerable ratio of nitrogen atoms in the conjugated core, the substitution pattern with a center of symmetry but lacking a plane of symmetry and large electron-rich/electron-deficient areas are promising for efficient charge transport.

**Figure 12:**
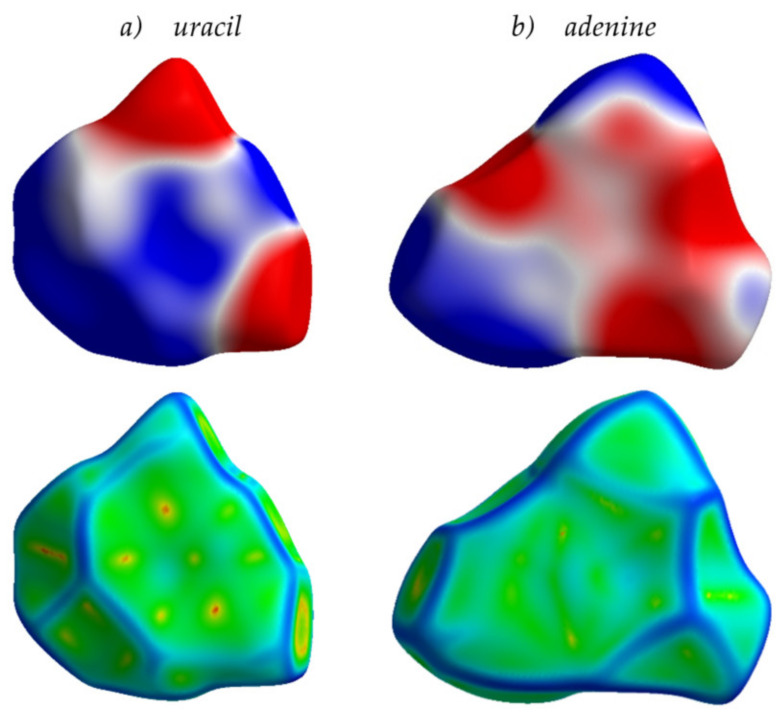
Hirshfeld surfaces for uracil (**a**) and adenine (**b**) crystals mapped with ESP (**top**) and curvedness (**bottom**). Details for the color scheme are given in Figure 5.

Finally, from the analysis provided herein, an interesting conclusion can be derived that goes beyond the frames of conventional organic electronics. Specifically, nitrogen-containing aromatic compounds are widespread in nature. For instance, nucleobases—the π-conjugated cores of the DNA and RNA monomer units—involve either purine or pyrimidine N-heterocycles. Multiple nitrogen heteroatoms in the nucleobases make the ESP of these compounds non-uniform, with pronounced areas of positive and negative charge (Figure 12). As a result, molecular packing for the crystalline nucleobases shows a significant amount of π-stacking like in the azaacenes addressed in this study. Indeed, as shown in Figure 12 for uracil and adenine, the Hirshfeld surfaces mapped with curvedness show flat green areas associated with π-stacking, in line with our conclusions for azaacenes (Figure 3). Charge transfer integrals for these crystals (which are actually wide-gap OSs [47]) are also rather large (up to 97 meV, Table S1). We suggest that the large *J* in these crystals stems from the favorable arrangement of the molecules due to the electrostatic interaction between the electron-rich and electron-deficient areas (ESP complementarity). These findings can be useful for the search and/or design of natural or nature-inspired OSs and can improve our understanding of charge transport in biomolecules, e.g., nucleic acids, which gained particular attention in the last decade due to its key role in bioprocesses [48,49]. A more detailed analysis of the intermolecular interactions and structure–property relationships in the assemblies of nucleobases is a subject of our ongoing study.

## 4. Materials and Methods

DFT calculations for isolated molecules were performed using the B3LYP density functional and 6-31*g*(d,p) basis set in the GAMESS package (Iowa State University, Ames, IA, USA) [50,51]. Geometry optimization was performed prior to all calculations. Reorganization energies and charge transfer integrals were calculated at B3LYP/6-31*g*(d,p) and B3LYP/6-31*g*(d) levels, respectively. Dispersion correction was not accounted for in these calculations since it affects only the energy and does not alter the electron density, which determines the properties addressed herein. Indeed, we observed that, for instance, *J* values were identical with and without Grimme’s D3 dispersion correction. The reorganization energy for charge transport, λ, was approximated by its inner sphere part, which is typically considered much larger than the outer sphere part [7]. The λ values for all the studied compounds were calculated according to the common potential energy surface (adiabatic potentials) scheme [7,10,34] from the energies of the molecule in the following four states: the neutral state in its optimized geometry (E_N_), the neutral state in the optimized geometry of the charged state (E_N_^*^), the charged state in its optimized geometry (*E_C_*) and the charged state in the geometry of the neutral state (E_C_^*^): λ = (E_N_^*^ − *E_N_*) + (E_C_^*^ − *E_C_*). For two compounds, **P** and **4NP**, *λ* values were also calculated using the normal mode decomposition method [7,52]. Charge transfer integrals *J* were calculated using a home-written code based on the dimer projection method (DIPRO) [53,54,55]. Raman spectra were calculated from static polarizabilities in the off-resonance regime.

Hirshfeld surface analysis and energy framework calculations were performed in CrystalExplorer17.5 software (The University of Western Australia, Perth, Australia) [56] at the B3LYP-D2/6-31 g (d,p) level. Note that, according to [57], using the D2 dispersion correction scheme in Crystal Explorer is preferable to using D3. For energy framework calculations, a molecular shell with a 3.8Å radius was generated around a central molecule, and the interaction energies (electrostatic, dispersion and total) between the molecular pairs were calculated. The scale factors for benchmarked energies used for the construction of energy models were taken from [58]. Crystal structures were obtained from the Cambridge Crystallographic Data Centre (CCDC) database; no further geometry optimization was performed. CCDC entry codes are listed in the Supplementary Materials (SM), Table S2.

## 5. Conclusions

To conclude, we have computationally analyzed the effect of the insertion of up to four nitrogen atoms (N-substitution) into the π-conjugated cores of anthracene, tetracene and pentacene. The relationship between the molecular structure, molecular properties, crystal structure and charge mobility was in the focus of the study. It has been found that N-substitution facilitates the π-stacking molecular arrangement in the corresponding crystals that generally results in large charge transfer integrals favorable for charge transport. The changes in molecular packing have been attributed to the dramatic changes in the molecular electrostatic potential, in line with earlier studies for lightly N-substituted oligoacenes. Hole and electron mobilities in the crystals of various azaacenes have been calculated for the first time, and their values exceeded 1 cm^2^ V^−1^ s^−1^ for several compounds. Importantly, azaacenes with four nitrogen atoms, forming pronounced electron-rich and electron-deficient areas arranged in a pattern with a center of symmetry but without a plane of symmetry, have shown either the largest electron or the largest hole mobilities in their series. Finally, we have extrapolated our conclusions on the nucleobases—natural compounds bearing multiple nitrogen atoms in their conjugated cores. Our results highlight that smart tuning of the molecular electrostatic potential can be an efficient tool in the design of organic semiconducting crystals for the steady improvement of organic electronics.

## Supplementary Materials

Supplementary materials can be found at https://www.mdpi.com/1422-0067/21/16/5654/s1.
