# Supplementary material for: Tuning of Molecular Electrostatic Potential Enables Efficient Charge Transport in Crystalline Azaacenes: A Computational Study
Source: Int J Mol Sci. 2020 Aug 6;21(16):5654. doi: 10.3390/ijms21165654 (PMC7460977; doi:10.3390/ijms21165654)
Supplement: Supplementary file 1 [file ijms-21-05654-s001.pdf]

International Journal of
Molecular Sciences

MDPI

Supplementary Materials

Tuning of Molecular Electrostatic Potential Enables Efficient Charge Transport in Crystalline Azaacenes: A Computational Study

Andrey Sosorev [1,2,*], Dmitry Dominskiy [3], Ivan Chernyshov [4] and Roman Efremov [1]

1 Department of Structural Biology, Shemyakin-Ovchinnikov Institute of Bioorganic Chemistry of the Russian Academy of Sciences, 117997 Moscow, Russia; r-efremov@yandex.ru
2 Molecular Spectroscopy Department, Institute of Spectroscopy of the Russian Academy of Sciences, 108840 Moscow, Russia
3 Faculty of Physics and International Laser Center, Lomonosov Moscow State University, 119991 Moscow, Russia; di.dominskiy@physics.msu.ru
4 ChemBio cluster, ITMO University, 191002 St.-Petersburg, Russia; ivan-chernyshoff@yandex.ru
* Correspondence: sosorev@physics.msu.ru

S1. Individual molecules: details

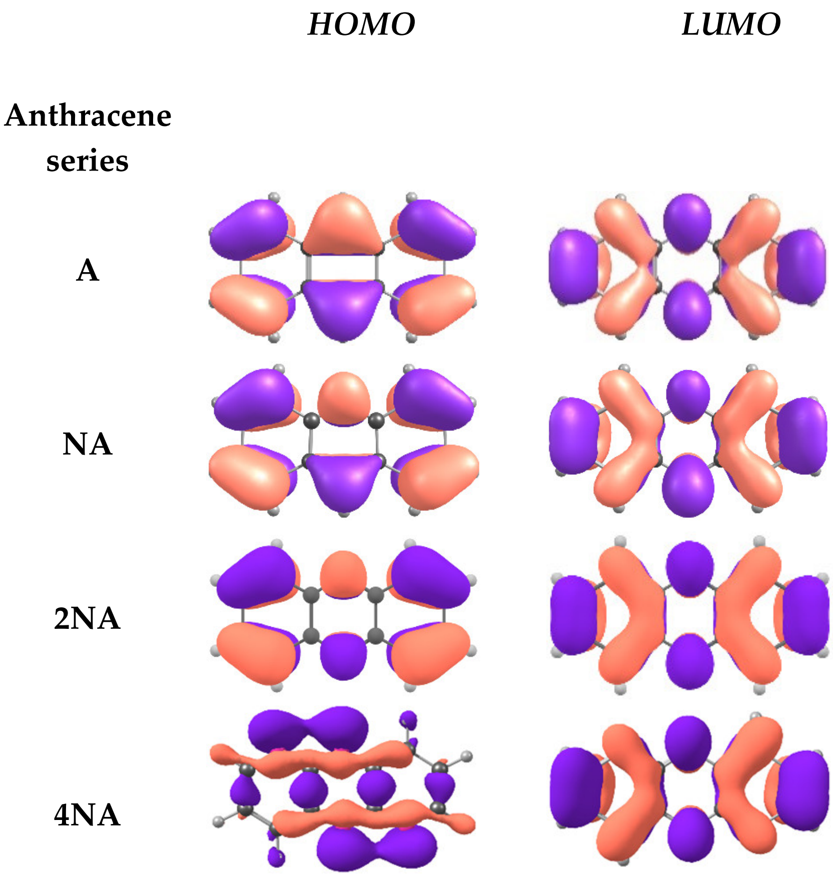

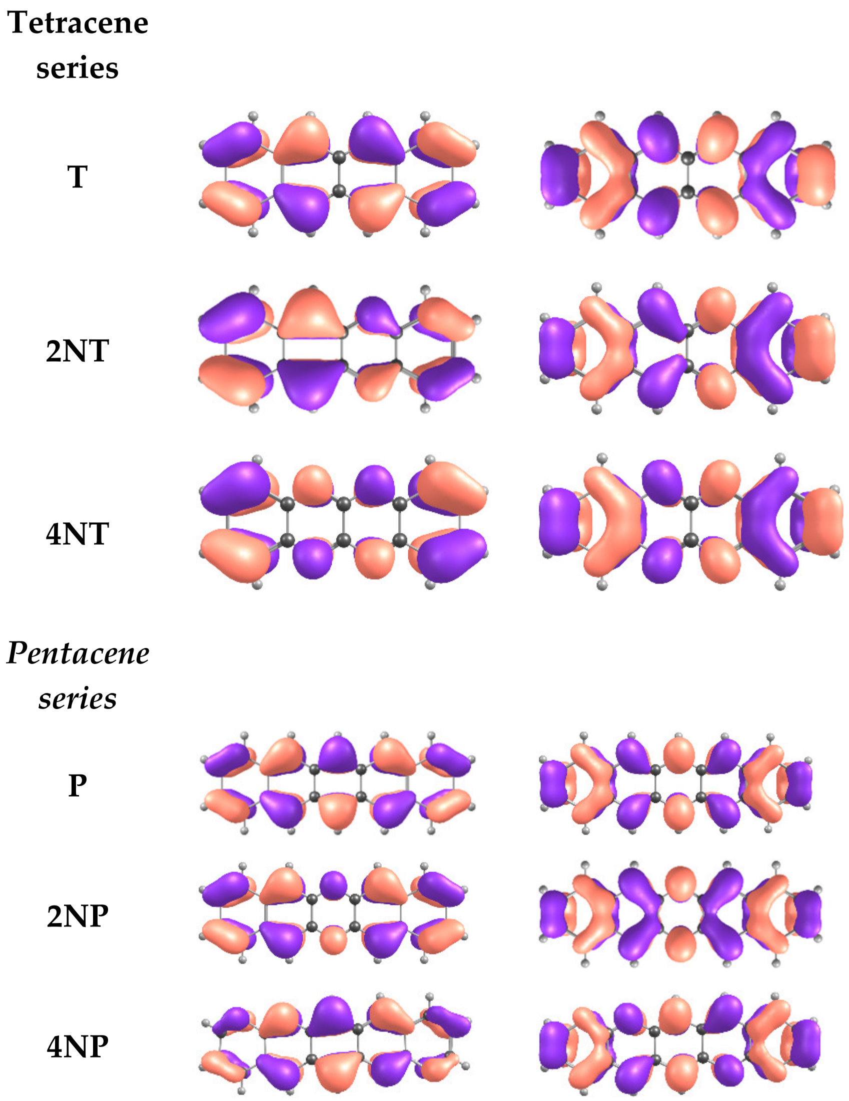

Figure 1. HOMO and LUMO patterns for the compounds studied.

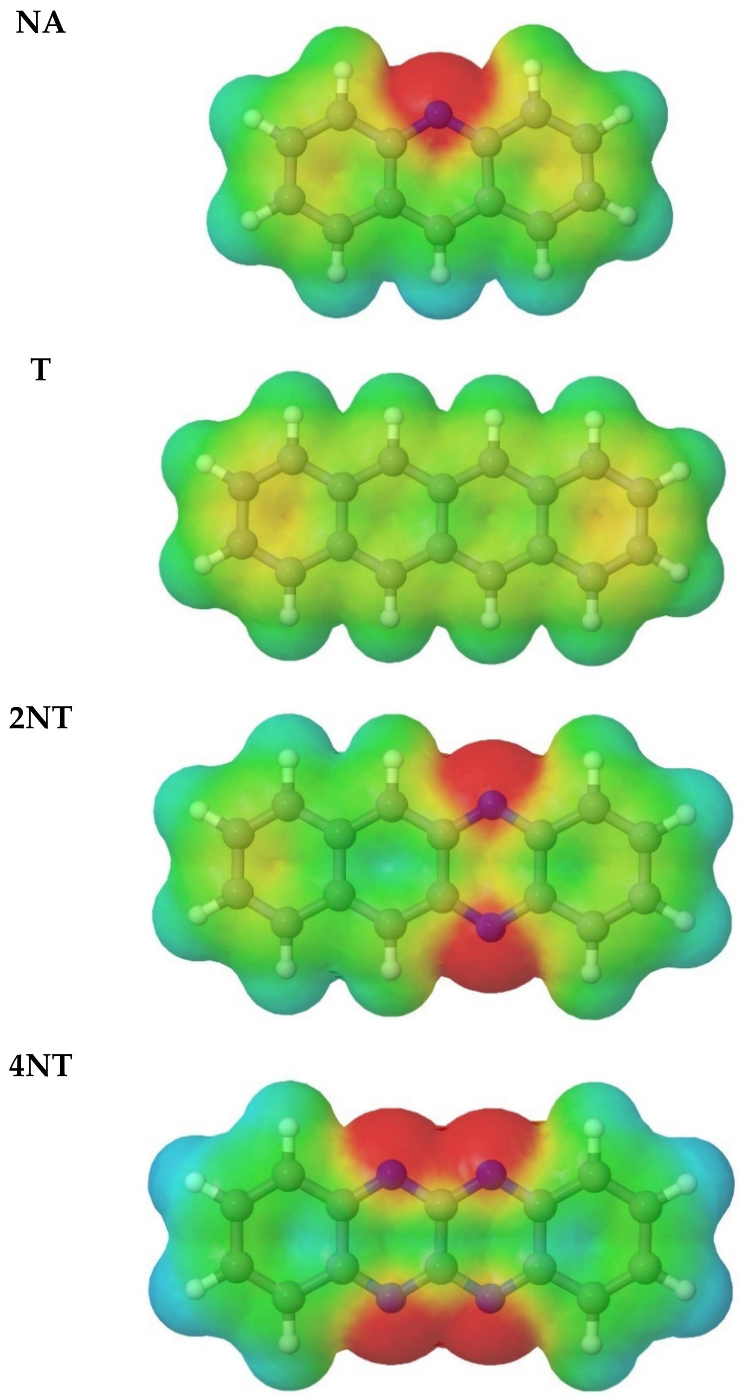

Figure 2. Molecular electrostatic potentials (ESPs) for the selected compounds studied.

S2. Crystal structure analysis details

Hirshfeld surfaces

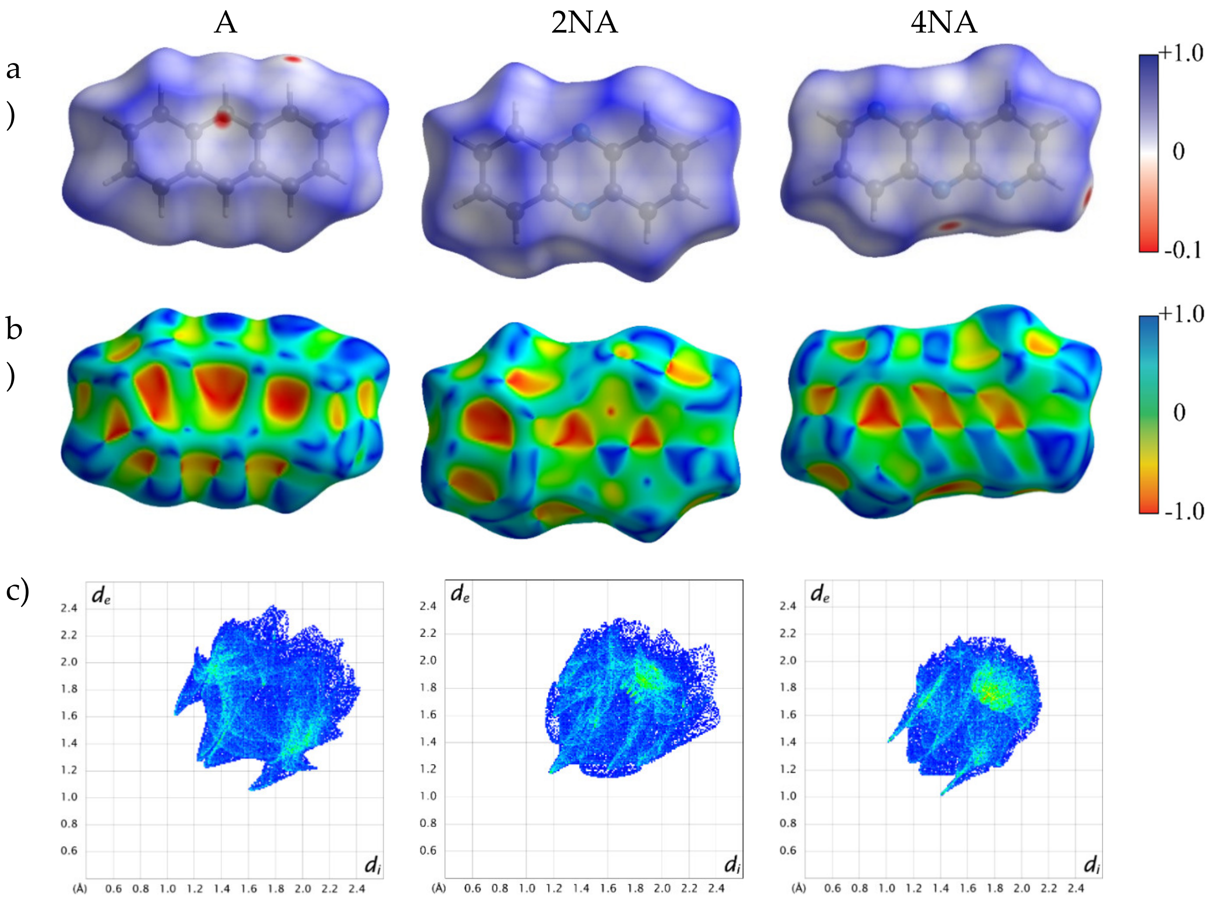

Figure 3. Hirshfeld surfaces of **A**, **2NA** and **4NA** mapped with normalized contact distance (a) and shape index (b). Red spots in (a) indicate intermolecular contacts closer than the sum of the van-der-Waals radii (close contacts), blue spots are referred to longer contacts, and contacts around the sum of van-der-Waals radii (moderate contacts) are white. 2D fingerprint plots (c) with d_i (distance from the atom inside the Hirshfeld surface) and d_e (distance from the atom outside the Hirshfeld surface) ranging from 0.5 to 2.9 Å. For any given d_i and d_e pairs, the change in color shows the raise in occurrence: white color for no occurrence, then blue green and red for the most frequent occurrence.

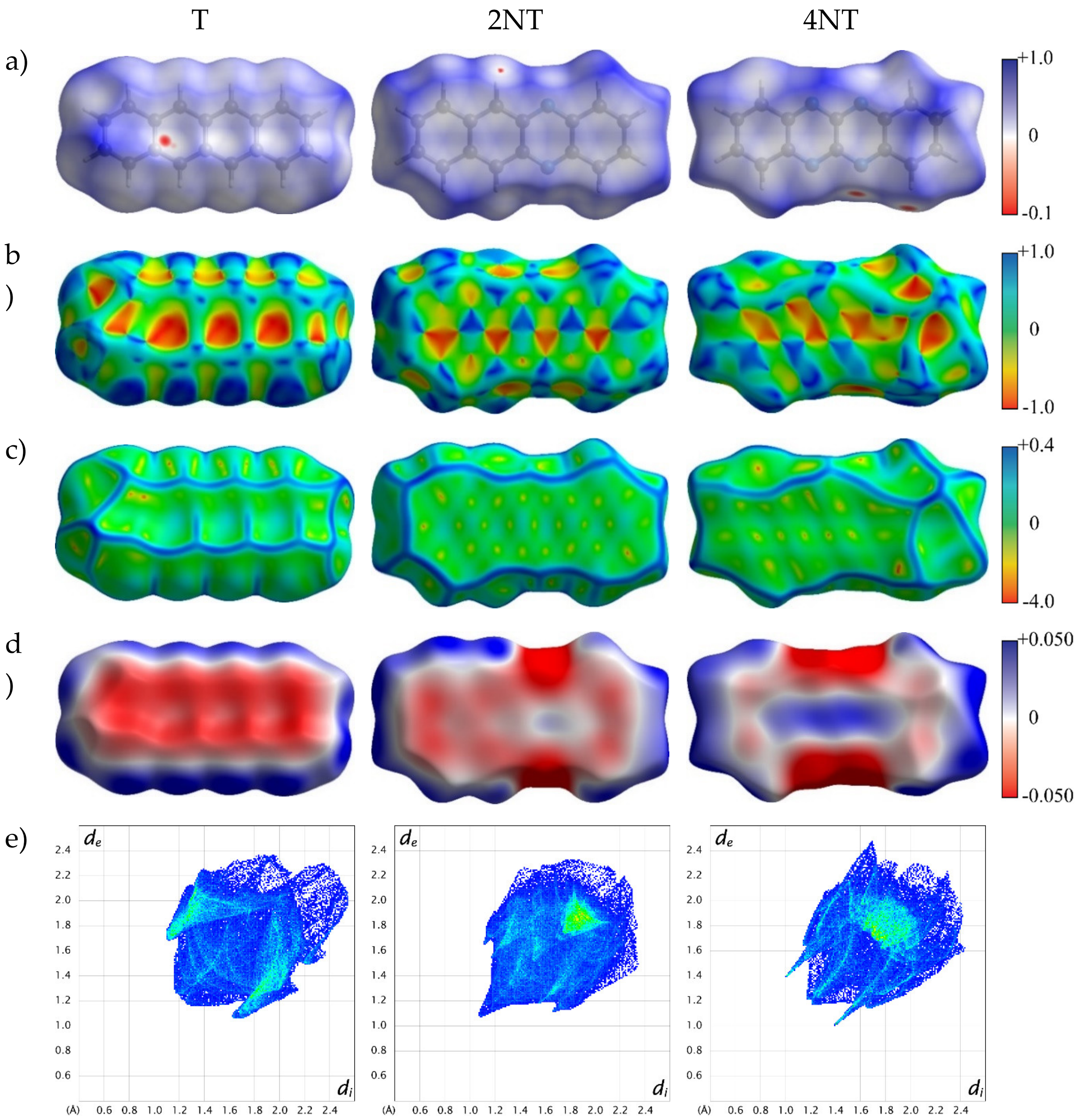

Figure 4. Hirshfeld surfaces for **T**, **2NT** and **4NT** mapped with normalized contact distance (a), shape index (b), curvedness (c) and ESP (±65.6 kJ mol^{-1} per unit charge) (d). Red spots in (a) indicate intermolecular contacts closer than the sum of the van-der-Waals radii (close contacts), blue spots are referred to longer contacts, and contacts around the sum of van-der-Waals radii (moderate contacts) are white. 2D finger print plots (e) with d_i and d_e ranging from 0.5 to 2.9 Å. For any given d_i and d_e pairs, the change in color shows the raise in occurrence: white color for no occurrence, then blue green and red for the most frequent occurrence.

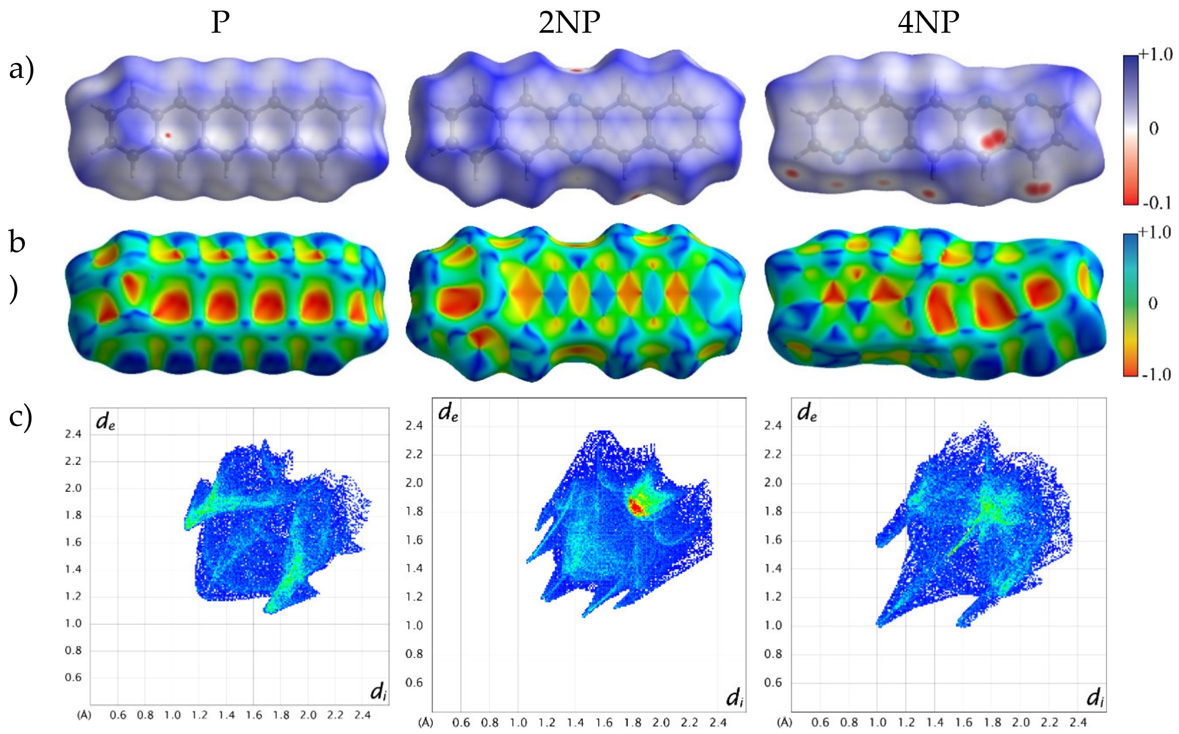

Figure 5. Hirshfeld surfaces for **P**, **2NP** and **4NP** mapped with normalized contact distance (a) and shape index (b). Red spots in (a) indicate intermolecular contacts closer than the sum of the van-der-Waals radii (close contacts), blue spots are referred to longer contacts, and contacts around the sum of van-der-Waals radii (moderate contacts) are white. 2D fingerprint plots (c) with d_i and d_e ranging from 0.5 to 2.9 Å. For any given d_i and d_e pairs, the change in color shows the raise in occurrence: white color for no occurrence, then blue green and red for the most frequent occurrence.

Energy frameworks

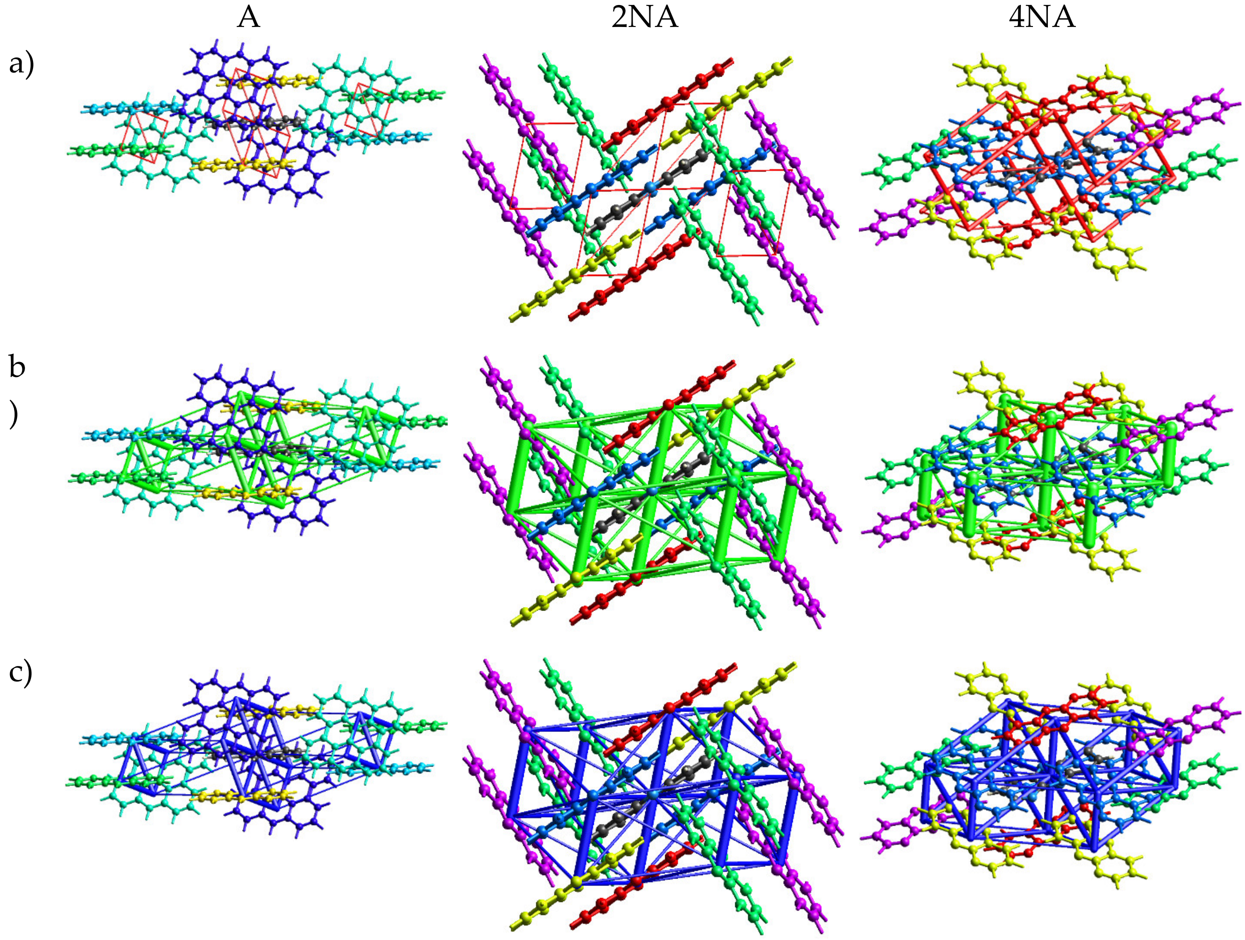

Figure 6. Graphical representation of intermolecular interactions (the Coulomb interaction energy in red on the panel a, the dispersion energy in green on the panel b, and the total interaction energy in blue on the panel c) in **A** (the first column), **2NA** (the second column) and **4NA** (the third column) crystals. The cylinders link molecular centroids, and their thickness is proportional to the magnitude of the energy; for clarity, pairwise energies with magnitudes less than 5 kJ mol^{-1} are omitted.

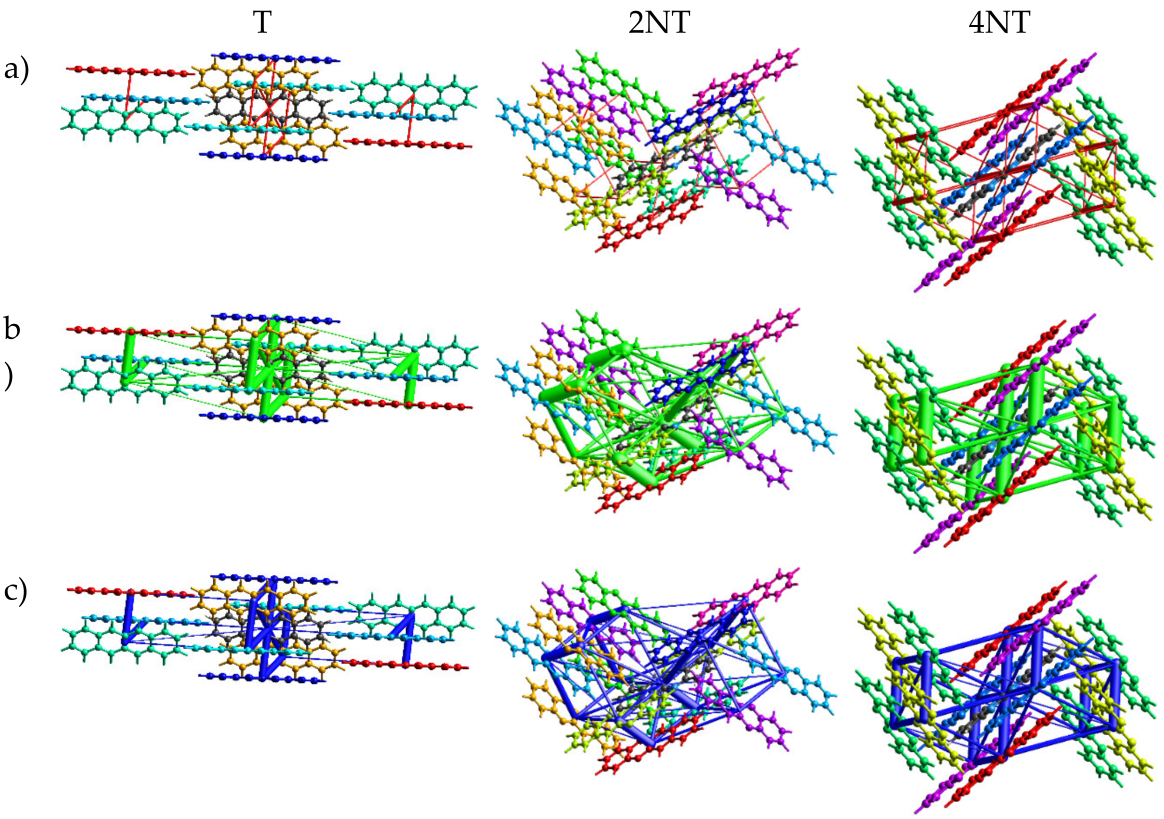

Figure 7. Graphical representation of intermolecular interactions (the Coulomb interaction energy in red on the panel a, the dispersion energy in green on the panel b, and the total interaction energy in blue on the panel c) in **T** (the first column), **2NT** (the second column) and **4NT** (the third column) crystals. The cylinders link molecular centroids, and their thickness is proportional to the magnitude of the energy; for clarity, pairwise energies with magnitudes less than 5 kJ mol^{-1} are omitted.

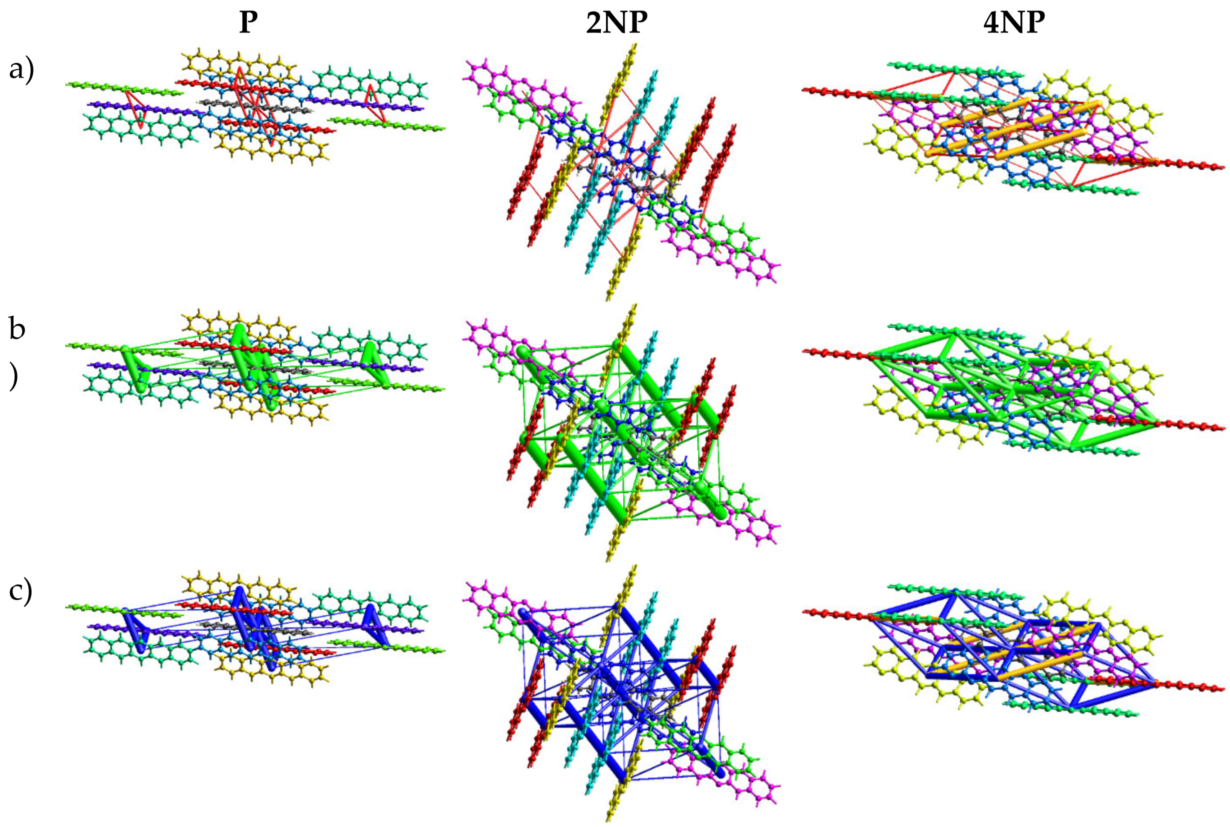

Figure 8. Graphical representation of intermolecular interactions (the Coulomb interaction energy: attraction in red and repulsion in yellow on the panel a, the dispersion energy in green on the panel b, and the total interaction energy in blue on the panel c) in **P** (the first column), **2NP** (the second column) and **4NP** (the third column) crystals. The cylinders link molecular centroids, and their thickness is proportional to the magnitude of the energy; for clarity, pairwise energies with magnitudes less than 5 kJ mol^{-1} are omitted.

Intermolecular contacts

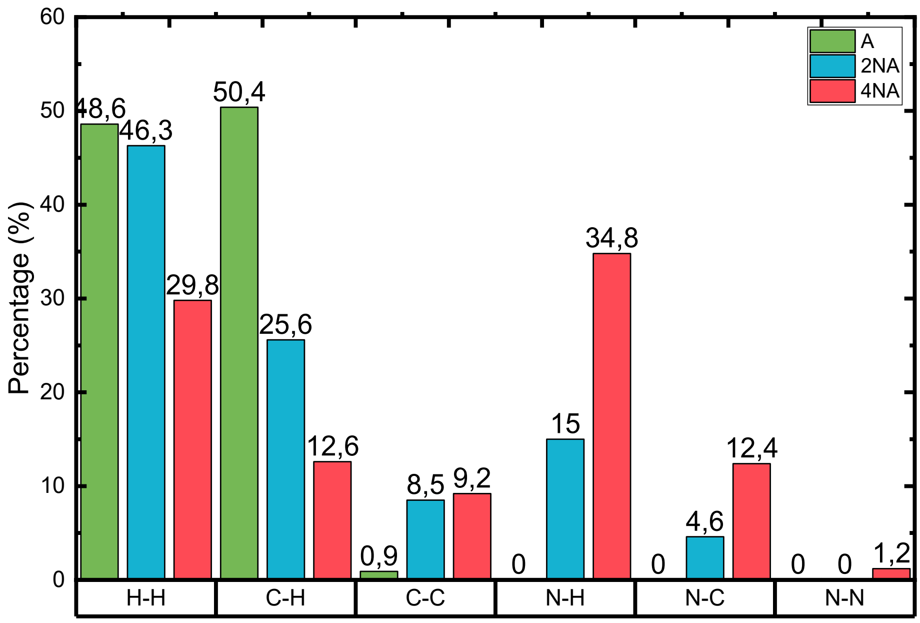

Figure 9. Distribution of reciprocal intermolecular contacts for **A**, **2NA** and **4NA** arranged by atom types. .

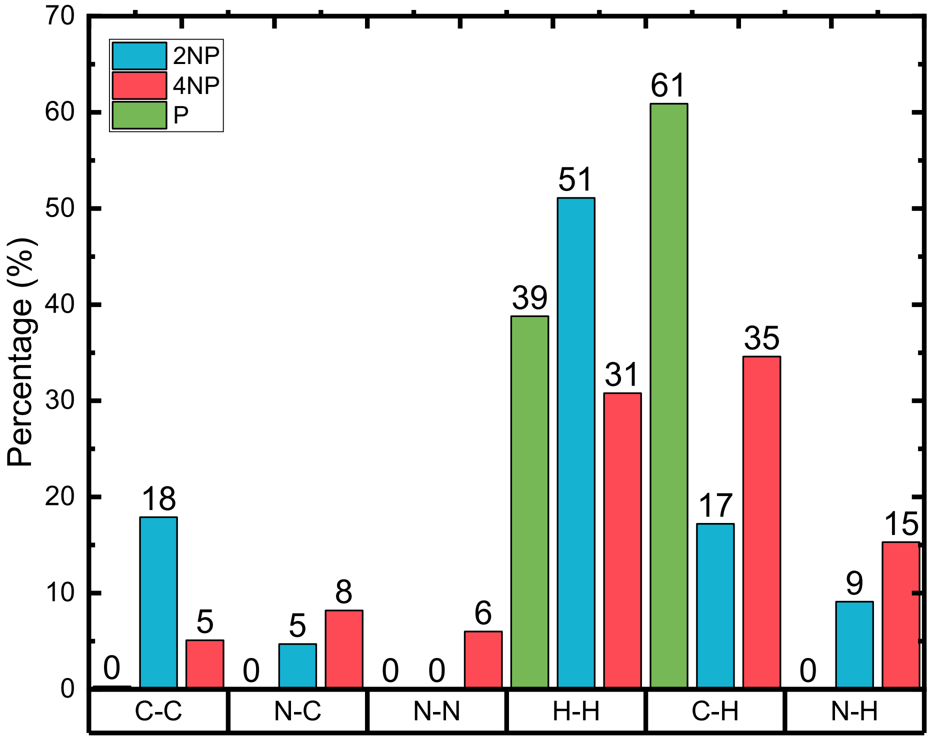

Figure 10. Distribution of reciprocal intermolecular contacts for **P**, **2NP** and **4NP** arranged by atom types.

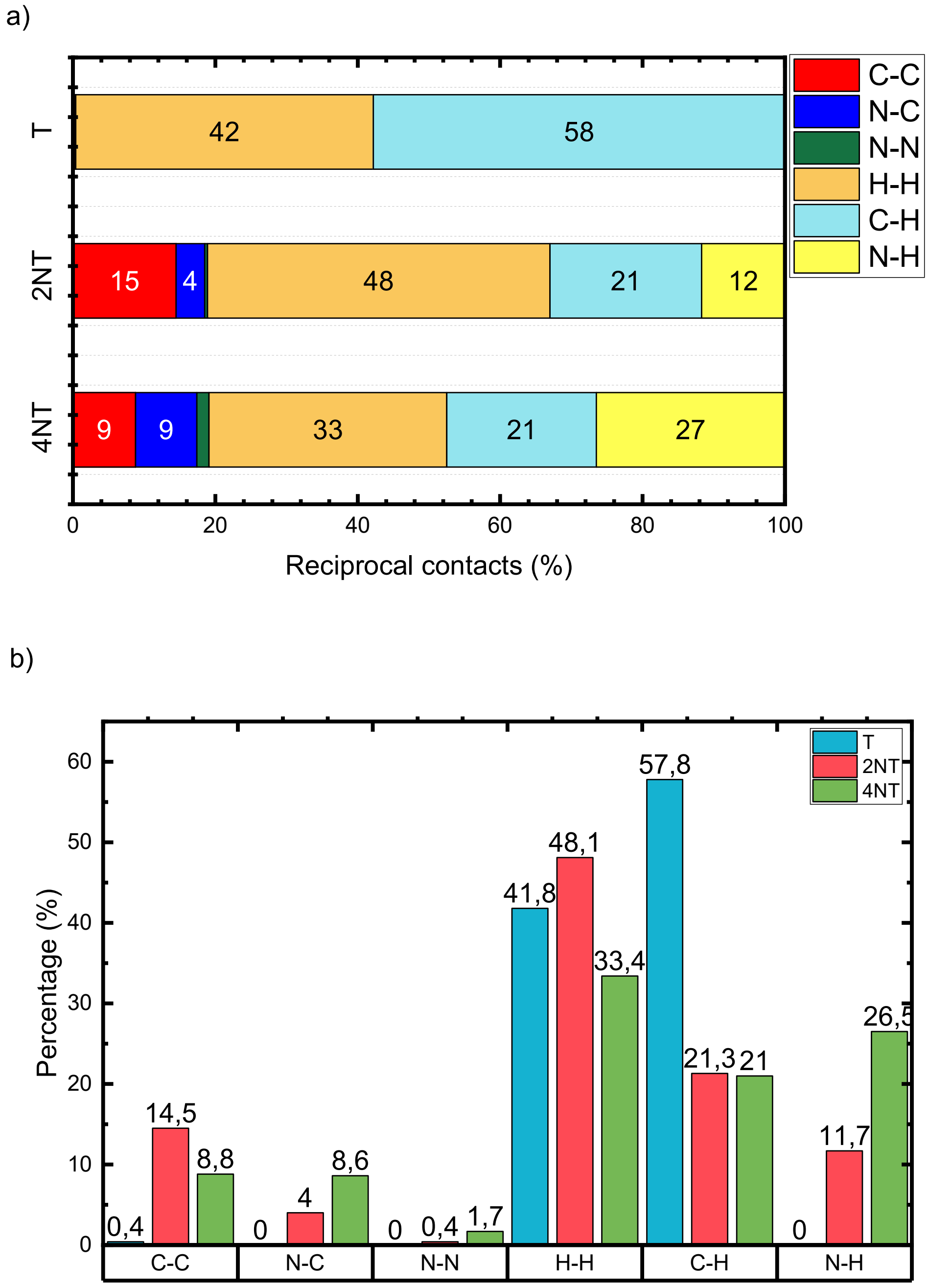

Figure 11. Distribution of reciprocal intermolecular contacts for **T**, **2NT** and **4NT** arranged by molecules (a) and atom types (b).

S3. Vibrations

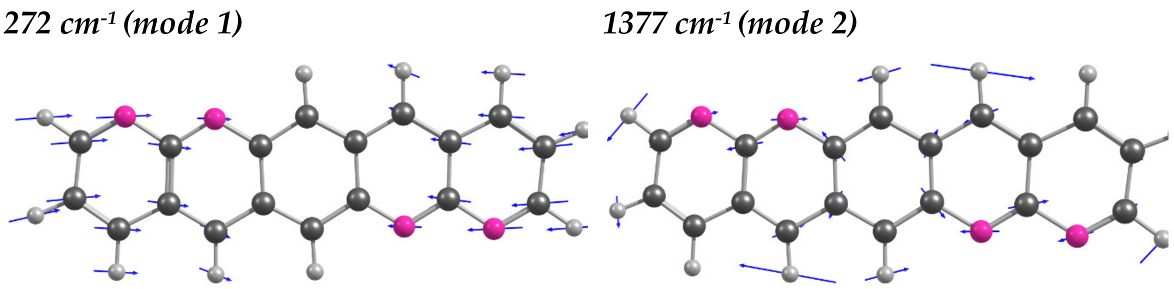

Figure 12. Vibrational displacements for the selected modes of 4NP.

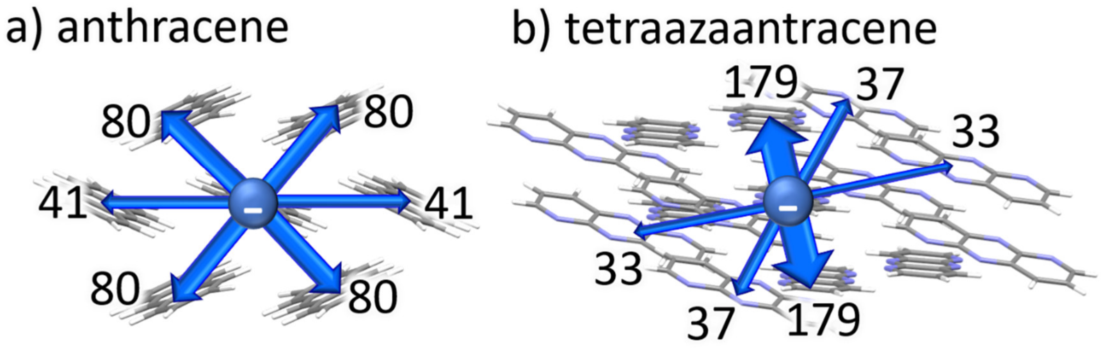

Figure 13. Electron transfer integrals for **A** and **4NA**.

S4. Charge mobility details

The charge-carrier diffusion coefficient was calculated by summation of the charge transfer rates k_i determined by Eq. (1) over all the transport directions, i.e. all the pairs of the given molecule with is neighbors:

$$D = \frac{1}{6}\sum_i k_i r_i^2 p_i, \quad \text{(S1)}$$

where r_i is the distance between the molecular centers along the *i*-th transport direction, and $p_i = k_i / \sum_j k_j$ is the probability for the charge to move in this direction. Finally, (isotropic) charge-carrier mobility was estimated using the Einstein–Smoluchowski relation:

$$\mu = \frac{eD}{kT} = \frac{e}{6kT}\sum_i k_i r_i^2 p_i. \quad \text{(S2)}$$

Table 1. Charge transfer integrals for uracil (left) and adenine (right) crystals.

**Uracil**				**Adenine**			
Dimer	*r*, A	J_h, meV	J_e, meV	Dimer	*r*, A	J_h, meV	J_e, meV
1	3.67	97	47	1	6.50	-11	-23
2	3.67	90	29	2	8.50	1	-1
3	7.57	-1	1	3	8.50	1	-1
4	6.27	-29	5	4	7.34	8	-46
5	6.95	12	-12	5	3.79	-61	-22
6	7.57	-1	1	6	3.79	-59	-20
7	6.27	-31	6	7	7.34	8	-44
8	6.95	13	-12	8	6.45	-8	-28
9	5.59	-4	25	9	10.14	0	0
10	5.99	5	-35	10	6.32	15	31
11	8.22	0	0	11	8.87	0	-0
12	7.37	-1	1	12	6.78	0	-13
13	6.37	31	-26	13	6.95	44	-15
14	6.15	15	27	14	8.84	0	-1
15	6.85	-5	-6	15	6.69	26	-53
16	6.85	-5	-6	16	6.79	49	-11
17	6.15	18	29	17	8.83	0	0

S5. Crystals: CCDC codes

Table 2. CCDC entry codes for the crystals studied.

**compound**	**Abbreviation**	**CCDC code**
anthracene	A	1103074
phenazine	2NA	1232385
dipyrido[2,3-b:2',3'-e]pyrazine	4NA	720329
tetracene	T	114446
benzo[b]phenazine	2NT	696053
quinoxalino[2,3-b]quinoxaline	4NT	297949
pentacene	P	114447
dibenzo[b,i]phenazine	2NP	N/A*
benzo[1,2-b:4,5-b']bis[1,8]naphthyridine	4NP	1866946

*obtained from Prof. Q. Miao.
